# The Relation of Alcoholism to Insanity, Pauperism and Crime

**Published:** 1902-09

**Authors:** Daniel R. Brower

**Affiliations:** Chicago, Professor of Nervous and Mental Diseases, Rush Medical College, etc.


### THE RELATION OF ALCOHOLISM TO INSANITY, PAUPERISM AND CRIME.*

BY DANIEL R. BROWER, M.D., LL.D., CHICAGO,

Professor of Nervous and Mental Diseases, Rush Medical College, etc.

In order to approach this question scientifically, we must consider the primary effects of alcohol on the human organization. We have stated elsewhere† "alcohol is classed popularly as a stimulant, and it does have this action in a transitory way, but its most striking effect is as an anesthetic, and a vasomotor depressant, producing degenerative changes in the neurons and connective tissue of the brain, and in the heart, arteries, liver and kidneys. The gross changes found in the nerve centers consist largely of neuritis, atheroma, fatty degeneration of blood-vessels, thickening of the membranes, inflammatory adhesions of the cortex and meninges, hemorrhagic foci, effusions into the ventricles, etc."

The profession are still disputing as to the therapeutic value of alcohol. Its use to-day is very limited, as compared with twenty years ago. Then it was considered impossible to carry to a successful termination a case of typhoid fever, pneumonia, and some other diseases without the use of alcoholics; now we frequently find in the practice of our leading physicians these and other cases successfully treated without the administration of a single dose of alcohol. That alcohol does stimulate the heart as a primary effect

---

* Read before the Georgia Sociological Society, Atlanta, June, 1902.

† The Manual of Insanity, W. B. Saunders & Co., 1902.

is admitted, and this stimulation forces blood to the brain out of proportion for its immediate necessities, and this undue and unnecessary increase of blood-supply to the brain must sometimes interfere with its proper functional activity, and may lay the foundation of pathological processes.

Now, we know that the brain requires for its proper functioning more blood than any other organ not engaged in blood-making and blood-purification, and the most delicate anatomical mechanism that the body possesses anywhere has been devised to keep the blood pressure in the brain uniform, and to properly distribute the blood to meet its varying requirements. These blood-vessels of the brain are surrounded by perivascular spaces, the function of which is to promptly remove from the cerebral tissue the waste material of cerebral activity, and the effect of alcohol is not only to increase the heart's action, but also to dilate the arterioles; and this increase in heart's action, and this dilatation of the arterioles must interfere with the regularity of the blood-supply, and, therefore, with the normal discharge of the cerebral neurons, and this is further intensified by the interference of the unduly dilated arterioles with the functions of the perivascular spaces.

Then, again, the brain requires a pabulum the most perfect that can be elaborated. Alcohol lessens the absorption of oxygen by the blood-corpuscles, and the exhalation of carbon dioxide; it diminishes the activity of the respiratory process in the lungs, and it produces degenerative changes in the liver and kidneys, in consequence of which the waste products of the body do not undergo normal metabolism; hence the blood is not properly purified, and the brain is supplied with blood below the normal standard, and the result is malnutrition.

Dr. H. J. Berkley * writes as follows of the manner in which the pathological lesions and the symptoms correspond one with another: "The sensory disorders, the exaggeration of the sensibility of the skin, the anesthetic troubles, and the ocular and auditory disorders would correspond to the beginning of the vascular disorders, when the nerve-cells, irritated by an insufficient supply of proper nutriment and excited by the presence of a poisonous

* Johns Hopkins Hosp. Reports, Vol. 6, 1897.

stimulus, overact for the time, and then as nutriment is still withheld from them altered metabolism results. The beginning swelling of the dendrites of the sensory motor organ is marked by paresthetic symptoms, those of the purer sensory organs by visual and ocular troubles, and some amnesia, especially for recent events; or, in other words, cells that have the function of evolving and transmitting thought cannot work properly, and defective memory results. Later, as the moter cells are more and more involved, and nuclear changes begin, continuous tremor becomes apparent, the muscles no longer coordinate perfectly, unless for a moment under the direct influence of the will. Still later, when a portion of the cell structures have become highly degenerated, and the altered cells have become more numerous, the already tottering will-power becomes more and more deadened, memory and judgment fail, aud when the degenerative process is far advanced an incomplete dementia is the final result."

These several effects of alcohol, especially if accompanied with a bad heredity and a bad environment, lead unerringly to insanity, pauperism and crime.

Maudsley,* writing upon this important question, states the case as follows: "Alcohol yields the simplest instance in illustration of the disturbing action on mind of a foreign matter introduced into the blood from without; here, where each phase of an artificially produced insanity is passed through successively in a brief space of time, we have the abstract and brief chronicle of the history of insanity. Its first effect is to produce an agreeable excitement, a lively flow of ideas, and a general activity of mind—a condition not unlike that which oftentimes precedes an attack of mania; then there follow, as in insanity, sensory and motor troubles and the automatic excitation of ideas which start up and follow one another without order, so that more or less incoherence of thought and speech is exhibited, while, at the same time, passion is easily excited, which takes different forms according to the individual temperament; after this stage has lasted for a time—in some longer, in others shorter—it passes into depression and maudlin melancholy, as convulsion passes into paralysis; the last scene of all be-

*Pathology of Mind, p. 194.

ing one of dementia and stupor. The different phases of mental disorder are compressed into a short period of time, because the action of the poison is quick and transitory; but we have only to spread the poisonous action over years, as the regular drunkard does, and we get a chronic and enduring insanity in which the foregoing scenes are more slowly acted. Or, if death, cutting short the career of the individual, puts a stop to the full development of the tragedy in his life, we may still have it played out in the lives of his descendants; since the drunkenness of the parent sometimes becomes the insanity of the offspring."

Dr. Bevan Lewis * writes: "Alcohol is a fertile source of nervous disease, and its implication of the nervous centers is so general and far-reaching that the resultant symptoms are of most protean nature; no poison, except the virus of syphilis, plays so extensive a rôle in the morbid affections and degeneration of the tissue, nervous and non-nervous."

The activity of alcohol as a causative factor in insanity is increasing. The 34th † annual report of the Crichton Royal Institute shows a rapid increase in insanity from drink, as follows: 1869, 8 per cent.; 1870, 16 per cent.; 1871, 23 per cent.; 1872, 29 per cent.; 1873, 35 per cent. Dr. Gilchrist remarks that doubtless a more minute analysis would largely increase the proportion of those in whom the excessive use of stimulants by the patients themselves, or by their parents, constitutes an important, if not the primary, factor in the production of mental disturbance.

Casper said that one-third of all patients in the Berlin pauper asylum were there from drink. At Bicêtre, M. Contease found one thousand cases of alcoholic insanity out of five thousand two hundred and thirty-eight cases.

A careful study of the subject in connection with the reports of American hospitals, and our own cases, tells the same sad story.

Alcohol diminishes the keenness of the moral sense; it blunts the acuity of discrimination between right and wrong, and it impairs the will-power, the power to do the right, and easily leads its victims into a vicious life.

---

*Text-book of Mental Diseases, p. 288.
† Psychological Medicine, Maun., p. 53.

Mr. Boris* writes: "Alcoholic drink is estimated to be the direct or indirect cause of 75 per cent. of all crimes committed, and of at least 50 per cent. of all the suffering endured on account of poverty in this country, and among civilized nations." And these observations of this eminent student of criminology do not differ from those who have made detailed studies in the same direction.

The increased consumption of alcohol is *pari passu* with the increase in crime. The number of criminals has increased 54.6 per cent. faster than the population during the last decade, and the increased consumption of alcoholics *per capita* of population has increased 53.9 per cent.

Fellows of the Georgia State Sociological Society, these are the facts, and it is for you to find the remedy. Such research and educational work as you have begun will be followed by mighty results in diminishing the increase of abnormality, not only in our own locality, but throughout the civilized world.

